# Successful treatment with daratumumab, lenalidomide, and dexamethasone therapy followed by autologous stem cell transplantation for newly diagnosed polyneuropathy, organomegaly, endocrinopathy, M-protein, skin changes syndrome: a case report

**DOI:** 10.1186/s13256-022-03552-y

**Published:** 2022-08-18

**Authors:** Ryutaro Taenaka, Sakurako Shimokawa, Ayako Katayama, Toshihiko Nagao, Teppei Obara, Naoaki Nishimura, Atsushi Tsujimoto, Kentaro Kohno, Kenichi Aoki, Ryosuke Ogawa

**Affiliations:** 1 Department of Hematology and Oncology, JCHO Kyushu Hospital, 1-8-1 Kishinoura, Yahatanishi-ku, Kitakyushu, Fukuoka Japan; 2 Department of Medicine and Biosystemic Sciences, Kyushu University Graduate School of Medicine, 3-1-1 Maidashi, Higashi-ku, Fukuoka City, Fukuoka Japan; 3 Department of Ophthalmology, JCHO Kyushu Hospital, 1-8-1 Kishinoura, Yahatanishi-ku, Kitakyushu, Fukuoka Japan; 4 Department of Dermatology, JCHO Kyushu Hospital, 1-8-1 Kishinoura, Yahatanishi-ku, Kitakyushu, Fukuoka Japan; 5 Department of Endocrinology and Metabolism, JCHO Kyushu Hospital, 1-8-1 Kishinoura, Yahatanishi-ku, Kitakyushu, Fukuoka Japan; 6 Department of Pharmacy, JCHO Kyushu Hospital, 1-8-1, Kishinoura, Yahatanisi-ku, Kitakyushu, Fukuoka Japan; 7 Department of Neurology, JCHO Kyushu Hospital, 1-8-1 Kishinoura, Yahatanishi-ku, Kitakyushu, Fukuoka Japan

**Keywords:** POEMS syndrome, DLd therapy, Autologous stem cell transplantation, Daratumumab, Lenalidomide

## Abstract

**Background:**

Transplant-eligible patients with polyneuropathy, organomegaly, endocrinopathy, M-protein, skin changes syndrome are treated with induction therapy and autologous stem cell transplantation. Conventional induction therapies may exacerbate neuropathy and a high rate of disease progression within 5 years. Furthermore, only 50% of patients are able to walk independently after the therapies. Daratumumab, lenalidomide, and dexamethasone therapy has been reported as a less neurotoxic, highly effective therapy for patients with polyneuropathy, organomegaly, endocrinopathy, M-protein, skin changes syndrome who are ineligible for transplant or whose syndrome is relapsed/refractory, but no reports have provided data from untreated transplant-eligible patients.

**Case presentation:**

A 34-year-old Japanese woman displayed weakness, pain and edema in the lower limbs, decreased grip strength, amenorrhea, and abdominal distention. She was unable to walk independently. The patient was diagnosed with polyneuropathy, organomegaly, endocrinopathy, M-protein, skin changes syndrome and performed four courses of daratumumab, lenalidomide, and dexamethasone therapy, which enabled her to walk independently and did not exacerbate the neuropathy. Hematopoietic stem cells were collected using plerixafor and filgrastim in combination. Autologous stem cell transplantation was performed with high-dose melphalan. At 3-month post-transplantation follow-up, most of her clinical symptoms had disappeared.

**Conclusions:**

Daratumumab, lenalidomide, and dexamethasone therapy followed by autologous stem cell transplantation may be more effective than conventional therapy for newly diagnosed polyneuropathy, organomegaly, endocrinopathy, M-protein, skin changes syndrome. Although there was concerns that daratumumab, lenalidomide, and dexamethasone therapy might lead to poor mobilization of hematopoietic stem cells, this was overcome with the combination of plerixafor and filgrastim. The benefit of daratumumab, lenalidomide, and dexamethasone as induction therapy prior to autologous stem cell transplantation should be confirmed in future clinical trials.

## Background

Transplant-eligible patients with polyneuropathy, organomegaly, endocrinopathy, M-protein, skin changes (POEMS) syndrome are treated with induction therapy and autologous stem cell transplantation (ASCT). Thalidomide and dexamethasone (Td) therapy [1] and lenalidomide and dexamethasone (Ld) therapy [2, 3] have been used as induction therapy. However, concerns have been raised that thalidomide may exacerbate neurological damage, and these therapies followed by ASCT have a high rate of disease progression within 5 years [1, 3]. Furthermore, only 50% of patients are able to walk independently after the therapies [3]. Daratumumab, lenalidomide, and dexamethasone (DLd) therapy has been reported as a less neurotoxic, highly effective therapy for patients with POEMS syndrome who are ineligible for transplant or those with relapsed/refractory POEMS syndrome [4, 5], but no reports have provided data from untreated transplant-eligible patients. In this report, we describe a case of newly diagnosed POEMS syndrome in which ASCT was performed after DLd therapy.

## Case presentation

**Fig. 1:**
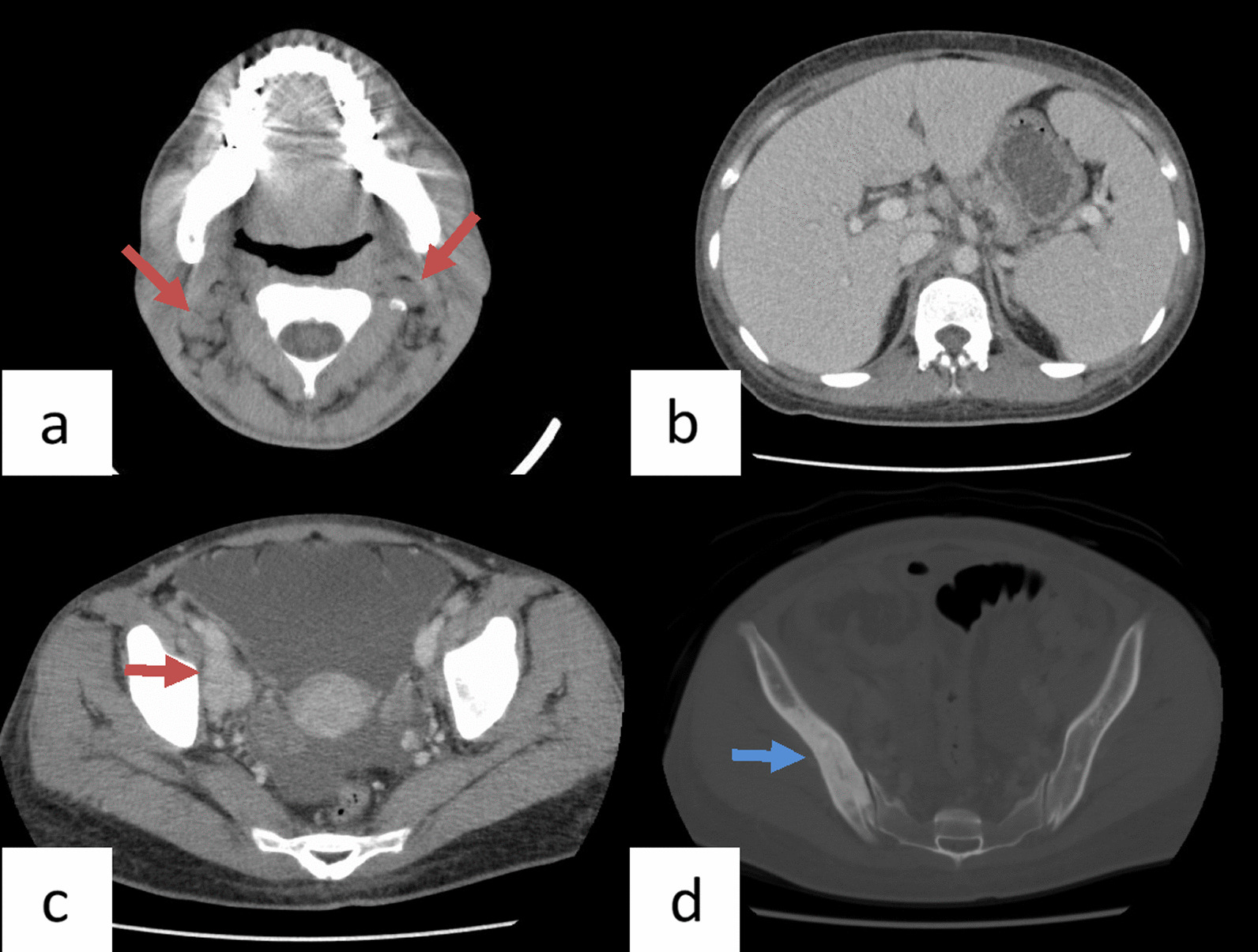
Computed tomography before treatment. **a** Cervical lymphadenopathy (reddish brown arrows); **b** hepatosplenomegaly; **c** right external iliac lymphadenopathy (reddish brown arrow) and ascites; **d** osteosclerosis of the ilium (blue arrow)

A 34-year-old Japanese woman displayed weakness, hypoesthesia, pain and edema (grade 1 according to National Cancer Institute Common Terminology Criteria for Adverse Events version 5.0) in the lower limbs, decreased grip strength, night sweats (grade 2), amenorrhea, abdominal distention, fatigue (grade 2), and weight loss of 10% in 6 months. Computed tomography (CT) revealed systemic lymphadenopathy, hepatosplenomegaly, ascites (grade 2), and multiple osteosclerosis (Fig. 1), and she was referred to our department on suspicion of a hematological disease.

**Fig. 2:**
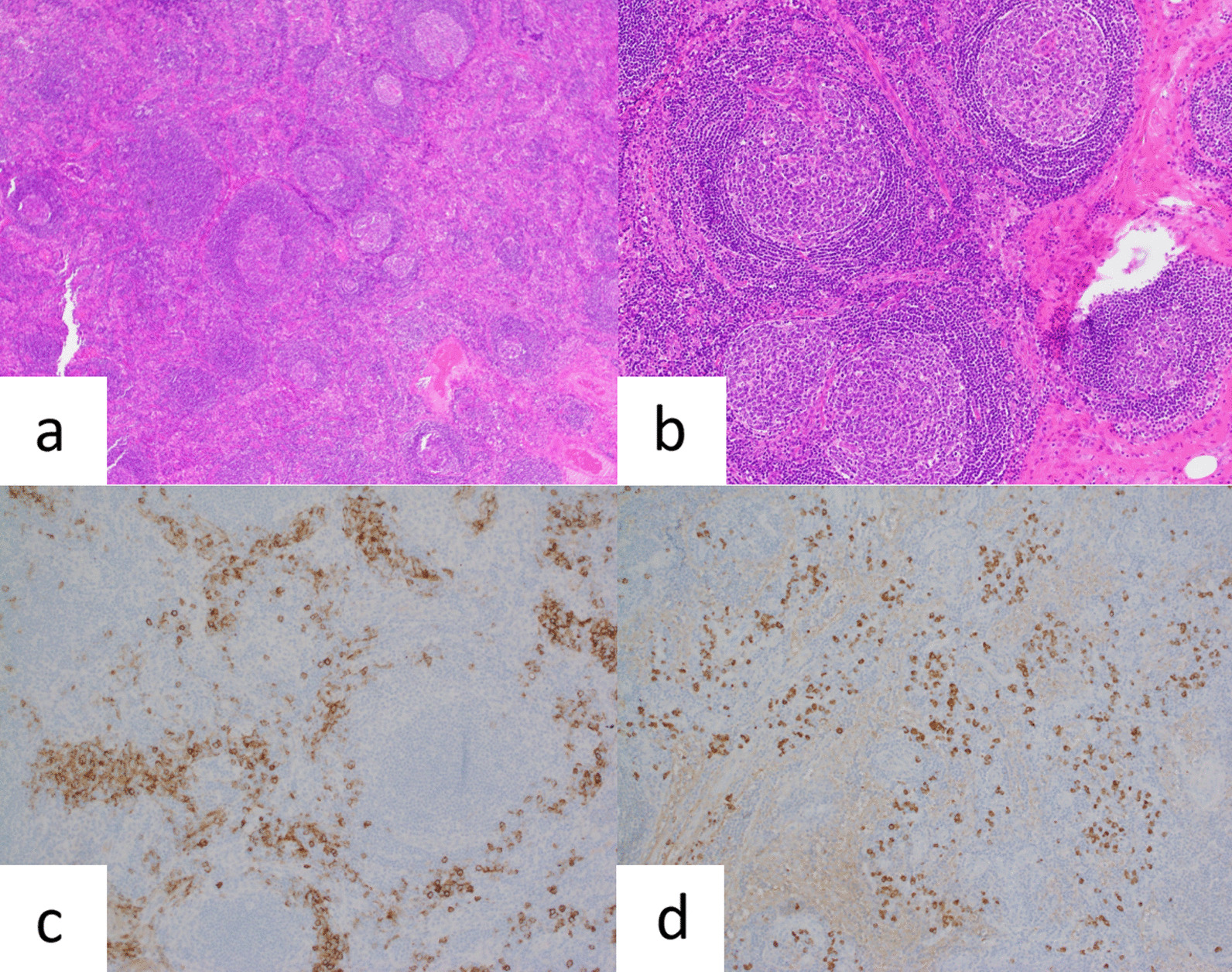
Cervical lymph node histopathology; multicentric mixed-type Castleman disease. Regressed germinal centers and proliferation of high endothelial venules with hyalinization. CD138 and immunoglobulin λ-positive plasma cells are seen in the interfollicular zones. **a**, **b** Hematoxylin–eosin staining; **c** immunohistochemical staining for CD138; **d** immunohistochemical staining for immunoglobulin λ

**Fig. 3:**
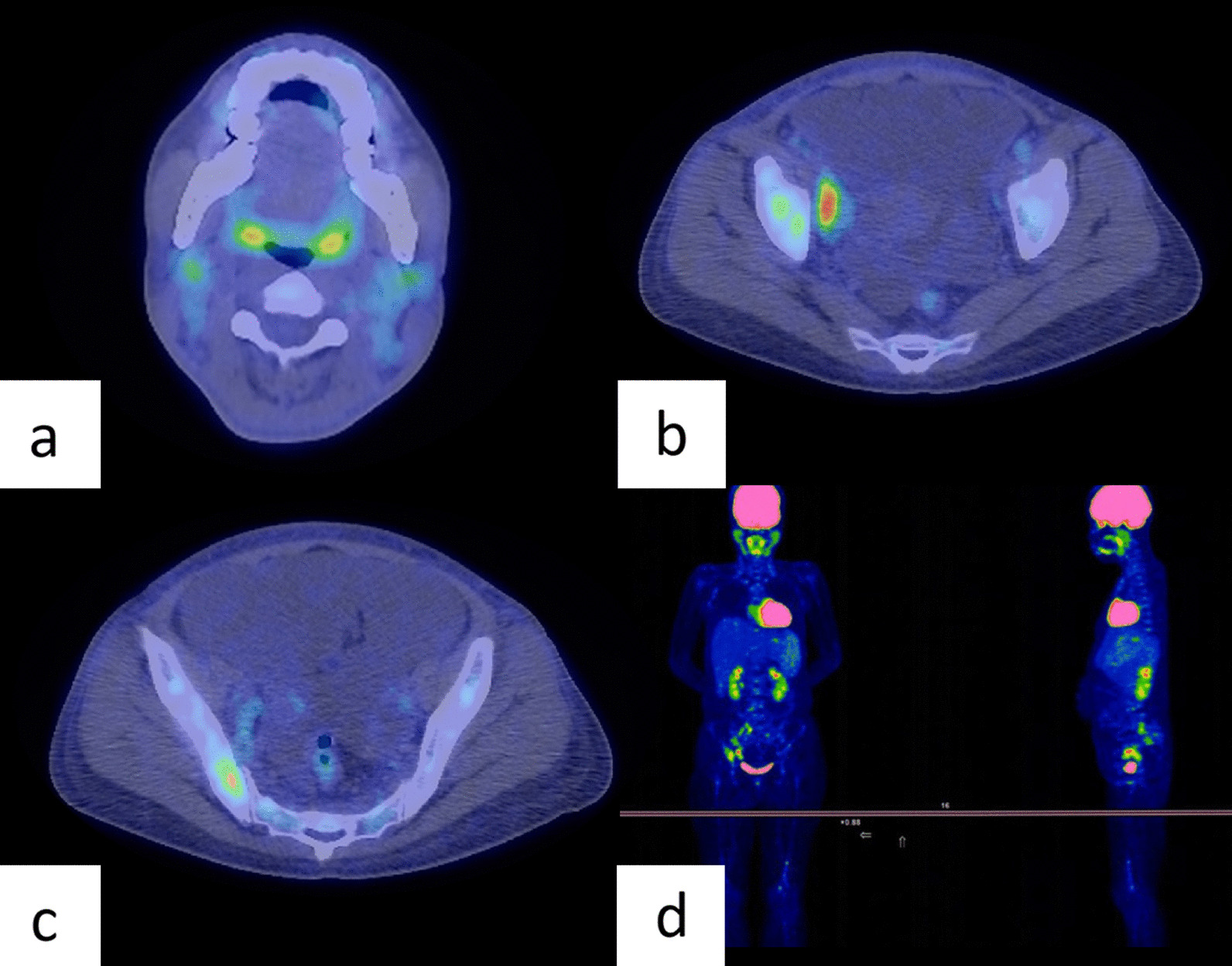
Fluorine-18-fluorodeoxyglucose positron emission tomography/computed tomography before treatment. **a** Cervical lymphadenopathy with FDG accumulation (SUV_max_ of 2.33); **b** right external iliac lymphadenopathy with FDG accumulation (SUV_max_ of 6.91); **c** osteosclerosis of the ilium with FDG accumulation (SUV_max_ of 4.16); **d** abnormal FDG accumulations in cervical, axillary, para-aortic, and pelvic lymphadenopathy and sclerotic lesions of the humerus, lumbar spine, ilium, and femur. FDG, fluorodeoxyglucose; SUV_max_, maximum standardized uptake value.

On admission, Eastern Cooperative Oncology Group performance status was 2. Overall Neuropathy Limitations Scale (ONLS) score was 2 for the arm grade and 3 for the leg grade (total score, 5). She was unable to walk independently and had to take a leave of absence. Peripheral sensory neuropathy and peripheral motor neuropathy were classified as grade 3. Nerve conduction velocity testing showed demyelinating polyneuropathy with a lower-extremity predominance. Serum electrophoresis and immunofixation showed a level of 1.81 g/dL for immunoglobulin A λ-type monoclonal protein. Bone marrow examination showed 3% plasma cells, and flow cytometry showed a λ bias. Human herpesvirus-8 deoxyribonucleic acid quantification in plasma was negative, and cervical lymph node biopsy revealed multicentric mixed-type Castleman disease (Fig. 2). Positron emission tomography–CT showed abnormal fluorodeoxyglucose (FDG) accumulations and a maximum standardized uptake value (SUV_max_) of 6.91, consistent with cervical, axillary, para-aortic, and pelvic lymphadenopathy and sclerotic lesions of the humerus, lumbar spine, ilium, and femur (Fig. 3). The serum level of vascular endothelial growth factor (VEGF) was 5000 pg/mL. Endocrine examination revealed central amenorrhea and subclinical hypothyroidism. Hyperpigmentation, hypertrichosis, and glomerular hemangiomas were observed. Papilledema (grade 1) and thrombocytosis were also observed. Platelet count was 447,000/μL. Respiratory function testing showed a diffusion capacity for carbon monoxide of 53%.

The diagnosis of POEMS syndrome is confirmed when both of the mandatory major criteria, one of the other three major criteria, and one of the six minor criteria are met [6]. Polyneuropathy (typically demyelinating) and monoclonal plasma cell-proliferative disorder (almost always λ) are mandatory major criteria. Castleman disease, sclerotic bone lesions, and VEGF elevation are other major criteria. Organomegaly (splenomegaly, hepatomegaly, or lymphadenopathy), extravascular volume overload (edema, pleural effusion, or ascites), endocrinopathy (adrenal, thyroid, pituitary, gonadal, parathyroid, pancreatic), skin changes (hyperpigmentation, hypertrichosis, glomeruloid hemangiomata, plethora, acrocyanosis, flushing, white nails), papilledema, and thrombocytosis/polycythemia are minor criteria. All mandatory major, other major, and minor criteria were present [6], so she was diagnosed with POEMS syndrome. After the patient provided written informed consent and approval was obtained from our institutional review board, 28-day cycles of DLd therapy (intravenous daratumumab at 16 mg/kg on days 1, 8, 15, and 22 during cycles 1 and 2, on days 1 and 15 during cycles 3 and 4; oral lenalidomide at 25 mg/day on days 1–21; and dexamethasone at 40 mg/day on days 1, 8, 15 and 22) were started. Symptoms began to improve during the first course and continued to improve over time. The only adverse events were diarrhea (grade 3) and a decrease in the white blood cell count (grade 1). Serum VEGF decreased to 1850 pg/mL and 826 pg/mL after one and four courses of DLd therapy, respectively. After four courses of DLd therapy, ONLS scores were 0 for the arm grade and 1 for the leg grade (total score, 1), allowing the patient to walk independently. All clinical symptoms and test results also improved (Table 1). Peripheral sensory neuropathy (grade 2) and peripheral motor neuropathy (grade 2) remained in the lower limbs.

**Table 1:** Laboratory values and clinical symptoms

	Before treatment	After four cycles of DLd	3 months after ASCT
Serum VEGF (pg/mL)	5000	826	212
Hematologic	1.81 g/dL M-spike in serum electrophoresis	0.50 g/dL M-spike in serum electrophoresis	Negative M-spike in serum electrophoresis, detectable M-protein in serum immunofixation
PET–CT	FDG-avid lesion SUV_max_ = 6.91	FDG-avid lesion SUV_max_ = 4.04	FDG-avid lesion SUV_max_ = 2.22
ONLS	5	1	0
MCV of median nerve (m/second)	36.1	36.6	43.5
Ascites	Grade 2	Grade 1	Absent
Edema	Grade 1	Absent	Absent
Papilledema	Grade 1	Grade 1	Absent
DLCO (%)	53	68.4	94.0
ECOG PS	2	1	0

DLd, daratumumab, lenalidomide, and dexamethasone; ASCT, autologous stem cell transplantation; VEGF, vascular endothelial growth factor; M-spike, monoclonal spike; M-protein, monoclonal protein; PET–CT, positron emission tomography-computed tomography; FDG, fluorodeoxyglucose; SUV, standardized uptake value; ONLS, Overall Neuropathy Limitations Scale; MCV, motor nerve conduction velocity; DLCO, diffusing capacity for carbon monoxide; ECOG PS, Eastern Cooperative Oncology Group performance status.

Hematopoietic stem cells were collected using plerixafor and filgrastim in combination (subcutaneous plerixafor at 0.24 mg/kg on days 3–4; subcutaneous filgrastim at 400 μg/m^2^ on days 1–5) at a dose of 3.7 × 10^6^ cells/kg of CD34-positive cells. ASCT was performed with high-dose melphalan (intravenous melphalan at 100 mg/m^2^ on days −3 and −2). As soon as neutrophils engrafted on day 10, fever of 40 °C with no identifiable infectious etiology, noncardiogenic pulmonary edema requiring oxygen administration at 2 L/minute by nasal cannula, and weight gain more than 2.5% of baseline body weight were identified. Engraftment syndrome was diagnosed, and steroid (intravenous methylprednisolone at 125 mg/day on days 10–12) was administered. Engraftment syndrome was quickly relieved, and the patient was discharged home on day 25. At 3-month post-transplantation follow-up, serum VEGF had decreased to 212 pg/mL and total ONLS score was 0. All clinical symptoms except central amenorrhea had disappeared, and all test results were improved (Table 1). The only residual neurological deficits were peripheral motor neuropathy (grade 1) and peripheral sensory neuropathy (grade 1) of the lower extremities. Peripheral neuropathies have continued to improve over time, and the patient is negotiating job conditions with her employer to return to work approximately 1 year after ASCT.

## Discussion and conclusions

The mainstay of induction is doublet therapy, such as Td therapy [1] and Ld therapy [2, 3]. However, in studies of ASCT after conventional induction therapy, the 5-year progression-free survival rate was only about 60% [1, 3]. Furthermore, only 50% of patients were able to walk independently at 24 months after ASCT [3]. Since the patient in our case was young and desired to return to work, we chose a triplet regimen that would be more effective than doublet therapy for achieving independent ambulation and long-term progression-free survival. Bortezomib [7] and thalidomide were avoided owing to the risks of neuropathy. DLd therapy was selected on the basis of the efficacy in patients with POEMS syndrome who are ineligible for or those with relapsed/refractory POEMS syndrome [4, 5]. In this case, the adverse events of DLd therapy were minor and four courses of DLd therapy enabled her to walk independently. DLd therapy did not exacerbate the neuropathy. Her symptoms continued to improve for a year after ASCT, and she was eventually able to return to work.

Lowering the serum level of VEGF below 1000 pg/mL before ASCT results in better 5-year clinical progression-free survival (90.9% versus 47.4%) and 5-year overall survival (100% versus 84.8%). On the other hand, hematological response before ASCT did not differ significantly in patients with relapse/progression after ASCT compared with those without relapse/progression [3]. These facts suggest that VEGF plays a central role in the pathogenesis of POEMS syndrome, and VEGF response, not hematological response, should be considered when selecting induction therapy. In the present case, serum VEGF was reduced to below 1000 pg/mL before ASCT, and a favorable prognosis was expected.

In transplant-eligible patients with POEMS syndrome, there is concern that daratumumab and lenalidomide may cause poor mobilization of hematopoietic stem cells [8, 9]. In the present case, a sufficient amount of autologous peripheral blood hematopoietic stem cells was collected with the combination of plerixafor and filgrastim.

Daratumumab is an antibody against CD38 that is predominantly expressed on plasma cells. CD38 is also expressed on immunosuppressive cells that suppress T cells, such as regulatory T cells, regulatory B cells, and myeloid-derived suppressor cells [10]. Daratumumab not only directly injured tumor cells, but also might have resulted in indirect attacks on tumor cells by activating the T cells by killing CD38-positive immunosuppressive cells. Lenalidomide might be highly effective against POEMS syndrome, not only inhibiting the growth of tumor cells, but also regulating the production of VEGF [11]. Furthermore, lenalidomide induces CD38 expression in plasma cells and immunosuppressive cells and might have enhanced the effects of daratumumab [12]. These mechanisms may be responsible for the favorable therapeutic effect of DLd therapy.

In this case, lymph node biopsy revealed Castleman disease. It has been reported that 11–30% of patients with POEMS syndrome have Castleman disease or Castleman disease-like tissue, and Castleman disease has been incorporated into the diagnostic criteria for POEMS syndrome [6]. In POEMS syndrome, cytokines secreted by monoclonal plasma cells are thought to cause Castleman disease, which is a polyclonal lymphoproliferative disease [13]. In POEMS syndrome with Castleman disease, treatment for POEMS syndrome has been performed with relatively good results [14]. In the present case, good treatment response was achieved with DLd therapy.

In conclusion, DLd therapy followed by ASCT may be more effective than conventional therapy for newly diagnosed POEMS syndrome. Although there were concerns that DLd therapy might lead to poor mobilization of hematopoietic stem cells, this was overcome with the combination of plerixafor and filgrastim. The benefit of DLd as induction therapy prior to ASCT should be confirmed in future clinical trials.

## Data Availability

The data that support the findings of this study are not publicly available owing to the presence of information that could compromise the privacy of the research participant, but are available from the corresponding author, Ryutaro Taenaka, upon reasonable request.

## Abbreviations

POEMS: Polyneuropathy, organomegaly, endocrinopathy, M-protein, skin changes
ASCT: Autologous stem cell transplantation
DLd: Daratumumab, lenalidomide, and dexamethasone
Td: Thalidomide and dexamethasone
Ld: Lenalidomide and dexamethasone
CT: Computed tomography
ONLS: Overall Neuropathy Limitations Scale
VEGF: Vascular endothelial growth factor
FDG: Fluorodeoxyglucose
SUV_max_: Maximum standardized uptake value

## References

[1] Nakaseko C. Autologous stem cell transplantation for POEMS syndrome. Clin Lymphoma Myeloma Leuk. 2014;14:21–3. 10.1016/j.clml.2013.12.009 24461808

[2] Li J, Huang X-F, Cai Q-Q, *et al*. A prospective phase II study of low dose lenalidomide plus dexamethasone in patients with newly diagnosed polyneuropathy, organomegaly, endocrinopathy, monoclonal gammopathy, and skin changes (POEMS) syndrome. Am J Hematol. 2018;93:803–9. 10.1002/ajh.25100 29603764

[3] Ohwada C, Sakaida E, Kawajiri-Manako C, *et al*. Long-term evaluation of physical improvement and survival of autologous stem cell transplantation in POEMS syndrome. Blood. 2018;131:2173–6. 10.1182/blood-2017-07-795385 PMC5946762 29599110

[4] Khan M, Stone K, van Rhee F. Daratumumab for POEMS syndrome. Mayo Clin Proc. 2018;93:542–4. 10.1016/j.mayocp.2018.02.001 29622102

[5] Gavriatopoulou M, Ntanasis-Stathopoulos I, Fotiou D, *et al*. Upfront daratumumab with lenalidomide and dexamethasone for POEMS syndrome. HemaSphere. 2020;4:e381. 10.1097/HS9.0000000000000381 PMC7306302 32647800

[6] Dispenzieri A. POEMS syndrome: 2017 update on diagnosis, risk stratification, and management. Am J Hematol. 2017;92:814–29. 10.1002/ajh.24802 28699668

[7] He H, Fu W, Du J, Jiang H, Hou J. Successful treatment of newly diagnosed POEMS syndrome with reduced-dose bortezomib based regimen. Br J Haematol. 2018;181:126–8. 10.1111/bjh.14497 28146276

[8] Eleutherakis Papaiakovou E, Terpos E, Kanellias N, *et al*. Impact of daratumumab-containing induction on stem cell mobilization and collection, engraftment and hospitalization parameters among multiple myeloma patients undergoing autologous stem cell transplantation. Blood. 2021;138:3886–3886.

[9] Kumar S, Dispenzieri A, Lacy MQ, *et al*. Impact of lenalidomide therapy on stem cell mobilization and engraftment post-peripheral blood stem cell transplantation in patients with newly diagnosed myeloma. Leukemia. 2007;21:2035–42. 10.1038/sj.leu.2404801 17581613

[10] Krejcik J, Casneuf T, Nijhof IS, *et al*. Daratumumab depletes CD38^+^ immune regulatory cells, promotes T-cell expansion, and skews T-cell repertoire in multiple myeloma. Blood. 2016;128:384–94. 10.1182/blood-2015-12-687749 PMC4957162 27222480

[11] Quach H, Kalff A, Spencer A. Lenalidomide in multiple myeloma: current status and future potential. Am J Hematol. 2012;87:1089–95. 10.1002/ajh.23234 22641420

[12] Fedele PL, Willis SN, Liao Y, *et al*. IMiDs prime myeloma cells for daratumumab-mediated cytotoxicity through loss of Ikaros and Aiolos. Blood. 2018;132:2166–78. 10.1182/blood-2018-05-850727 30228232

[13] Fajgenbaum DC, Shilling D. Castleman disease pathogenesis. Hematol Oncol Clin N Am. 2018;32:11–21. 10.1016/j.hoc.2017.09.002 29157613

[14] Dispenzieri A. POEMS syndrome: 2019 update on diagnosis, risk-stratification, and management. Am J Hematol. 2019;94:812–27. 10.1002/ajh.25495 31012139

